# Subcutaneous immunoglobulin therapy for adult patients with primary immunodeficiency disease: Qatar experience

**DOI:** 10.5339/qmj.2023.sqac.3

**Published:** 2023-11-19

**Authors:** Sherin Thalappil, Sally Khalil, Slim Hassini, Maryam Al-Nesf

**Affiliations:** ^1^Adult Allergy and Immunology Section, Department of Medicine, Hamad Medical Corporation, Doha, Qatar. Email: SThalappil@hamad.qa

## Abstract

Introduction: Subcutaneous immunoglobulin (SCIG) and intravenous immunoglobulin (IVIG) are used for the treatment of primary immunodeficiency (PIDD). SCIG is as effective as IVIG in preventing infections.^1^ However, SCIG has advantages over IVIG as it causes fewer systemic reactions and can be infused at home by the patient leading to improved quality of life.^2^

Methods: We retrospectively analyzed adult patients with PIDD who received SCIG in an Adult Allergy Clinic in Qatar. Patients who received IVIG before SCIG and are naïve to IgG replacement were included. We compared Serum IgG levels, the number of antibiotic courses received, and the number of hospital admissions one year before and one year after starting SCIG. SF36 score was used to compare health-related quality of life scores before SCIG and after one year of SCIG.

Results: Twenty patients were included in the study, of which 17 were on prior IVIG replacement, and three were naive to replacement. SCIG replacement resulted in the maintenance of serum IgG levels in those who received IVIG prior. SCIG resulted in a statistically significant reduction in the number of antibiotics prescribed and hospitalization in the naïve subgroup but no substantial change in the prior IVIG group. 6/20 patients developed side effects like injection site pain, swelling, and headache. No patients developed significant systemic side effects. 10/20 patients discontinued the SCIG therapy, four patients due to side effects, and others due to noncompliance and financial reasons. SF36 Score was compared for the five patients in IVIG prior group and showed no significant improvement in individual score but improvement in the overall score (p=0.003)

Conclusions: In our experience, SCIG therapy effectively prevents recurrent infection in PIDD patients, and patients did not experience any significant systemic side effects. There is a substantial improvement in the quality of life. However, compliance continues to be a problem.

## Figures and Tables

**Table 1.** Patient demographics, clinical features, and results of SCIG treatment.

**Demographics**	**IVIG Naïve (n=3)**	**IVIG prior (n=17)**	**P value**
Age at SCIG initiation, mean± SD (range) in years	28.5±13.8 (15-65)	40.6±13 (28-54)	
SCIG dose [g/month] (range)	44.70(20-64)	35.33(28-48)	
Sex			
Female	7 (41%)	2(67%)	
Male	10(59%)	1(33%)	
Nationality			
Qatari	11(65%)	2(67%)	
Non-Qatari	6 (35%)	1(33%)	
Diagnosis			
CVID	5(29%)		
SAD	2(12%)	1(33.33%)	
Hypogammaglobulinemia	4(23.5%)	1(33.33%)	
Agammaglobulinemia	4(23.5%)		
IgG subclass deficiency	1(6%)		
ALPS	1(6%)	1(33.33%)	
Complications			
Bronchiectasis, rhinosinusitis	11(65%)	2(67%)	
Comparison pre and post-SCIG	Pre SCIG	Post SCIG	
Average Serum IgG level (g/L) for 1 year in Naïve group	4.146	10.126	0.1176
Average Serum IgG levels (g/L) for one year in IVIG prior group	9.7155	10.0245	0.3091
Antibiotic prescription (in number per year) for one year in the naïve group	5	1.33	0.0315
Antibiotic prescription (in number per year) for one year in IVIG prior	0.73	0.27	0.2708
Number of hospitalizations (in number per year) for one year in naïve	3	0	**0.0351**
Number of hospitalizations (in number per year) for one year in IVIG prior	3.64	1.73	1.91
